# The Story of the Insane from Year to Year

**Published:** 1906-11-24

**Authors:** 


### THE STORY OF THE INSANE FROM YEAR TO YEAR.

EIGHTEENTH ANNUAL SERIES.

### AYR DISTRICT ASYLUM.

Here the numbers fell during the year from 508 to 497. There were 151 admissions; 61 recoveries, and 68 deaths. The total number under treatment was 659, and the average daily number resident was 499. The recoveries were 42 per cent. of the admissions, and the deaths were 13.62 per cent. of the average resident; both of these percentages being above the average of similar institutions. Of the year's deaths, 19 were caused by cerebral and spinal diseases; 12 by consumption, and 10 by other lung diseases. Of the year's admissions 72 cases owed their insanity to hereditary tendency, and 33 to alcoholic excess. Dr. Easterbrook divides the latter into three classes. First, the true dipsomaniac who seldom finds his way into asylums, who is screened by his friends, and may sometimes be induced to go into inebriate homes. Second, the careless or ignorant drinker who constantly takes too much, until some day a breakdown occurs. This type forming a large proportion of the alcoholic insanity met with in asylums. Third, the alcoholic deteriorate, or the chronic inebriate, who has spent his money, mind, and morals in drink, and is left as a derelict for the asylum. At this stage he is usually beyond redemption. Dr. Easterbrook looks back on a "quiet year," and neither seclusion nor restraint was used. The weekly cost per head was 10s. 6d.

